# Update of the WHO global air quality guidelines: Systematic reviews – An introduction

**DOI:** 10.1016/j.envint.2022.107556

**Published:** 2022-12

**Authors:** Román Pérez Velasco, Dorota Jarosińska

**Affiliations:** World Health Organization (WHO) Regional Office for Europe, European Centre for Environment and Health, Platz der Vereinten Nationen 1, 53113 Bonn, Germany

## Abstract

This paper aims to serve as an introduction to the Special Issue in *Environment International* entitled “Update of the WHO Global Air Quality Guidelines: Systematic Reviews”. The article has two main objectives. One is to provide the context to this Special Issue, related to (a) policy context, overall exposure to air pollution, and burden of disease attributable to air pollution, and the other is to describe (b) the WHO guideline development process, with special emphasis on the systematic reviews. In particular, this paper presents the systematic reviews and other supporting evidence that was used and discussed during the process and summarizes important methodological information about the approaches taken to conduct the systematic reviews. These approaches include the definition of population, exposure, comparator, outcomes and study design (PECOS) questions, the assessment of the risk of bias in individual studies and the assessment of the overall certainty of the evidence. In summary, the new WHO global air quality guidelines are informed by the best available scientific evidence covering a vast number of research papers published until September 2018, and appraised by experts and stakeholders in the field of air quality. However, research gaps remain and, therefore, further research is warranted.

## 1 Background

Air pollution is the greatest environmental threat to health and well-being at the global scale, and all populations are affected to a different extent (WHO, 2016a). Air pollution consists of a mixture of substances in the atmosphere that can cause harm to humans, other living organisms and the environment, including at low concentrations. Of major concern is air pollution originating from combustion of fossil fuels and biomass (Vallero, 2014, WHO Regional Office for Europe, 1980, WHO Regional Office for Europe, 2006). A growing body of evidence indicates that air pollution is responsible for a range of adverse health outcomes, affecting multiple organs and systems of the human body (Bazyar et al., 2019, Kelly and Fussell, 2015, Who, 2018a). In addition, some air pollutants, such as ground-level ozone (O_3_) and black carbon, are also short-lived climate pollutants in that they affect the climate system (Campbell-Lendrum and Prüss-Ustün, 2019, Who, 2015).

## 2 Policy context

While WHO has been working on air quality and health for decades, it was in May 2015 that 194 WHO Member States endorsed the resolution *Health and the environment: addressing the health impact of air pollution.* The document states the need to intensify the efforts of Member States and WHO to protect populations from the health risks of air pollution (World Health Assembly, 2015). In response to the resolution and the roadmap that followed (WHO, 2016b), the first WHO Global Conference on Air Pollution and Health took place in October 2018. Conference participants recognized the need to tackle air pollution, and set the aspirational goal of reducing attributable deaths by two thirds by 2030 (WHO, 2018b, Who, 2018a).

Likewise, the United Nations Environment Assembly (UNEA) has adopted three resolutions on the topic in recent years, highlighting the health effects of all forms of air pollution, including dust and sandstorms, as well as the importance of cross-sectoral action and international cooperation (Unea, 2016, Unea, 2017a, Unea, 2017b).

At the broader United Nations (UN) level, the 2030 Agenda for Sustainable Development includes both explicit and implicit connections between 10 of the Sustainable Development Goals (SDGs) and air pollution (Longhut et al., 2018, Unga, 2015). In particular, efforts to combat air pollution are instrumental to achieving SDG 3 on good health and well-being, SDG target 7.2 on access to clean energy in households, SDG target 11.6 on air quality in urban settlements, SDG target 11.2 on access to sustainable transport and SDG 13 on climate action (WHO, 2018b, Who, 2018a).

Closely linked with the 2030 Agenda, the 2018 Political Declaration of the Third High-Level Meeting of the UN General Assembly on the Prevention and Control of Non-Communicable Diseases (NCDs) explicitly recognizes exposure to air pollution as one of the five key risk factors of NCDs, alongside physical inactivity, poor diet, alcohol consumption and tobacco use (UNGA, 2018). A 2019 report from the UN Special Rapporteur on the issue of human rights obligations relating to the enjoyment of a safe, clean, healthy and sustainable environment emphasizes Member States’ obligations to ensure the right of all people, in particular vulnerable groups, to breathe clean air (UNGA, 2019).

## 3 Exposure to air pollution

Governments typically track levels of air pollutants and estimate exposure by deploying fixed-site monitoring station networks (WHO Regional Office for Europe, 1999). Yet, many jurisdictions lack that capacity, and existing systems are often tracking a limited number of pollutants, such as particulate matter (PM) (Awe et al., 2017, Cromar et al., 2019, Unep, 2017).

To bridge these gaps and raise awareness on air pollution, WHO compiles global databases on ambient and household measurements. In particular, the WHO Ambient Air Quality Database includes annual mean concentrations of PM with a diameter ≤ 2.5 µm (PM_2.5_) and ≤ 10 µm (PM_10_), as well as nitrogen dioxide (NO_2_), from more than 6000 human settlements in over 100 countries. Although the Database includes data from both high-income countries (HICs) and low- and middle-income countries (LMICs), challenges remain in obtaining data from the latter (WHO, 2022a). In turn, the Global Database of Household Air Pollution Measurements presents data on household air pollution microenvironments, methods and measurements. The dataset, representing 46 countries, covers household and personal air pollution data (e.g., characteristics of microenvironments, exposure measurements for PM and carbon monoxide) (WHO, 2018c, Who, 2018a).

Collectively, these measurements are used by WHO as inputs to run global predictive modelling of both ambient and household PM_2.5_ exposures, using advanced statistical techniques and data from complementary sources. Modelled exposure data are more comprehensive than ground-based measurements, and allow the calculation of the health impacts and burden of disease from air pollution for all Member States and territories (Shaddick et al., 2018). Using these data, WHO also monitors and reports progress in the air pollution exposure and health impact indicators of the SDGs (7.1.2 and 11.6.2; and 3.9.1, respectively) (WHO, 2022a).

Based on the most recent data, the population of only 10 % of the assessed settlements was exposed to annual mean levels of PM_2.5_ or PM_10_ aligned with the WHO global air quality guidelines (WHO, 2021). For NO_2_, the population of only 23 % of the assessed settlements was exposed to annual mean levels aligned with the WHO guidelines (WHO, 2022b).

## 4 Burden of disease attributable to air pollution

Research conducted in many regions of the world indicates that air pollution is associated with an increasing variety of adverse health effects. Effects of air pollution have been documented both in the short term, such as exacerbations of disease leading to premature mortality or hospital admissions, and in the long term, including cardiovascular, respiratory, oncologic, neurologic, metabolic, psychiatric and birth outcomes (Bazyar et al., 2019).

WHO estimated that the global harm attributable to ambient PM_2.5_ and household air pollution in 2016 was equivalent to 4.2 and 3.8 million deaths, respectively, while the combined effects of air pollution amounted to 7 million deaths in the same year. Most of this burden is born by LMICs, with the WHO South-East Asia Region being the most affected, followed by the Western Pacific and African regions. Looking into specific causes of death, ischaemic heart disease was responsible for 34 % of the joint estimates of mortality, while acute lower respiratory infections, stroke, chronic obstructive pulmonary disease and lung cancer accounted for 21 %, 20 %, 18 % and 7 %, respectively (WHO, 2018d, Who, 2018a, WHO, 2018e, WHO, 2018f).

Similarly, economic burden estimates are very high. A study conducted by the World Bank and the Institute of Health Metrics and Evaluation (IHME) reported that air pollution cost the global economy approximately US$ 225 billion in 2013, leading to US$ 5.11 trillion in welfare losses and US$ 225 billion in lost labour income (World Bank and IHME, 2016).

Although progress to reduce these burdens has been made in many parts of the world, current trends in urbanization and ageing, together with the ongoing epidemiological transition towards NCDs, highlight the urgency of making renewed efforts to reduce air pollution worldwide (West et al., 2016, Who, 2009).

## 5 WHO air quality guidelines

Since the 1980s, WHO has produced several volumes of air quality guidelines, both for ambient and indoor air. Although the approaches to developing them have evolved over time, WHO air quality guidelines are widely viewed as the global reference to protect human health from the effects of air pollution (WHO Regional Office for Europe, 2017).

In particular, the *WHO air quality guidelines, global update 2005* was used as a basis to inform the setting of policies and standards in a wide range of jurisdictions. Focusing on the so-called classical pollutants of PM_2.5_, PM_10_, O_3_, NO_2_ and sulfur dioxide (SO_2_), these guidelines include thorough risk assessments and recommended levels, as well as interim targets to gradually reduce air pollution (Krzyzanowski and Cohen, 2008, WHO Regional Office for Europe, 2006).

While recommendations on other ambient pollutants are available in the previous volumes of air quality guidelines for Europe (WHO Regional Office for Europe, 1987, WHO Regional Office for Europe, 2000), WHO has also issued dedicated global indoor air quality guidelines since 2009, including on dampness and mould (WHO Regional Office for Europe, 2009), selected chemicals (WHO Regional Office for Europe, 2010), and household fuel combustion (WHO, 2014a).

## 6 New WHO global air quality guidelines: The systematic reviews

Since the publication of the 2005 global update, there has been a substantial increase in findings from many new studies that have strengthened the evidence base for established pollutant − outcome pairs and identified associations with a broader number of health outcomes, as documented in recent WHO evaluations such as the *Review of evidence on health aspects of air pollution – REVIHAAP Project* (WHO Regional Office for Europe, 2013) and the *Air pollution and cancer* reports of the International Agency for Research on Cancer (IARC) (Cohen et al., 2013).

In light of the new evidence, especially at the lower range of the exposure distribution, the scientific community recommended the development of new air quality guideline levels. WHO Member States echoed this call for updated air quality guidelines in 2015, recognizing their utility as an effective instrument to help decision-makers confront the air pollution problem (World Health Assembly, 2015).

Guided by the *WHO handbook for guideline development* (WHO, 2014b), the new WHO global air quality guidelines have been developed under the oversight of the WHO European Centre for Environment and Health (ECEH). Informed by the six systematic reviews of adverse health effects from air pollution published in this issue (Whaley et al., 2021), a number of recommendations in the form of air quality guideline levels were formulated. These recommendations are accompanied by complementary guidance in the form of interim targets and good practice statements.

In this endeavour, several groups of experts and stakeholders holding different roles and responsibilities worked closely together. These included the WHO steering group, the systematic review team (SRT), the guideline development group (GDG) and the external review group (ERG). To address biases, internal mechanisms to prevent potential conflict of interests and expert advice on procedural and methodological aspects were provided.

In its meetings, the GDG decided to formulate air quality guideline levels, with an indication of the shape of the concentration − response relationships, for the classical pollutants PM_2.5_, PM_10_, O_3_, NO_2_, SO_2_ and carbon monoxide; interim targets; and qualitative advice in the form of good practice statements for specific types of PM, namely black carbon/elemental carbon, ultrafine particles, and particles originating from sand and dust storms.

In a similar fashion, health outcomes upon which air quality guideline levels were developed for each air pollutant were prioritized according to several criteria, including determinations of causality from reputed scientific bodies, outcome severity, contribution to burden of disease, policy relevance and future exposure trends. Overall, the outcomes considered critical for decision-making by the GDG were all-cause mortality, cause-specific mortality (due to lung cancer, respiratory diseases and cardiovascular diseases), and emergency department visits and hospital admissions due to asthma and myocardial infarction.

Using the population, exposure, comparator, outcome and study design (PECOS) model (Morgan et al., 2018), the following key questions were formulated to search the literature and identify relevant studies:

• *In any population, including subgroups of susceptible adults and children, what is the increase in risk of a health outcome per unit increase in µg/m^3^ of long-term exposure (in the order of months to years) to ambient concentration (both outdoor and indoor) of the air pollutant, observed in studies relevant for the health outcome and exposure duration of interest?*
- *In these studies, what is the lowest concentration that produces a measurable increase in risk?*
• *In any population, including subgroups of susceptible adults and children, what is the increase in risk (incidence or prevalence) of health outcome per unit increase in µg/m^3^ of short-term exposure (in the order of hours to days) to ambient concentration (both outdoor and indoor) of an air pollutant, observed in studies relevant for the health outcome and exposure duration of interest?*
• *In these studies, what is the lowest concentration that produces a measurable increase in risk?*

Taken together, WHO commissioned the following systematic reviews of evidence on the critical health outcomes associated with both long- and short-term exposure to the selected air pollutants:

• Long-term exposure to PM and all-cause and cause-specific mortality: a systematic review and *meta*-analysis (Chen and Hoek, 2020; this issue);
• Long-term exposure to O_3_ and NO_2_ and all-cause and cause-specific mortality: a systematic review and *meta*-analysis (Huangfu and Atkinson, 2020; this issue);
• Short-term exposure to particulate matter (PM_2.5_ and PM_10_), nitrogen dioxide and ozone and all-cause and cause-specific mortality: systematic review and *meta*-analysis (Orellano et al., 2020; this issue);
• Short-term exposure to sulfur dioxide and all-cause and respiratory mortality: a systematic review and *meta*-analysis (Orellano et al., 2021; this issue);
• Short-term exposure to ozone, nitrogen dioxide, and sulfur dioxide and emergency room visits and hospital admissions due to asthma: a systematic review and *meta*-analysis (Zheng et al., 2021; this issue); and
• Short-term exposure to carbon monoxide and myocardial infarction: a systematic review and *meta*-analysis (Lee et al., 2020; this issue).

All systematic reviews were based on a common protocol as recommended by the *WHO handbook for guideline development*, which was later fine-tuned to address the specific question of each review. The individual protocols were registered by the SRT on PROSPERO, an international register of systematic reviews maintained by the University of York in the United Kingdom (Page et al., 2018).

The instruments needed to assess risk of bias and the overall certainty of the evidence were adapted to better reflect the particularities of the air quality and health field. These modifications were piloted before their application and are briefly described below. More detailed descriptions are published by WHO (WHO Global Air Quality Guidelines Working Group on Risk of Bias Assessment, 2020) and in the appendices of the systematic reviews in this issue (WHO Global Air Quality Guidelines Working Group on Certainty of Evidence Assessment, 2020).

To assess risk of bias of individual studies, a specific instrument was developed by a working group composed of GDG members and methodologists. Based on a review of existing tools, the group agreed to consider six key domains (confounding, selection bias, exposure assessment, outcome measurement, missing data, selective reporting), each including several subdomains or signalling questions. The group also prepared guidance notes to assist the SRT in performing the task, including a list of critical and additional potential confounders and key information on the particularities of air pollution exposure assessment. To avoid carrying forward the ratings from one domain to the others, the working group proposed conducting subgroup analyses per risk of bias domain across studies, grouping studies at higher and lower risk of bias for that domain. This approach was considered more suited than an overall judgment of bias at the study level (Morgan et al., 2019).

To assess the overall certainty in the bodies of evidence, another working group adjusted the standard Grading of Recommendations, Assessment, Development and Evaluation (GRADE) approach. GRADE is the most widely used tool to assess clinical interventions, because it offers a transparent and structured framework that separates judgments about certainty in the epidemiological evidence from other considerations. Different groups have made efforts to adapt GRADE for the assessment of evidence on exposures in recent years, but consensus does not yet exist (Morgan et al., 2016). Unlike other adaptations, this was not aimed to assess causality through an examination of all the relevant streams of research (Woodruff and Sutton, 2011), but to rate how certain one could be that the true estimate of the association between an air pollutant and an adverse health effect lied in a range (Hultcrantz et al., 2017).

Consistent with the standard approach, the certainty of the effect estimate is graded as high, moderate, low or very low (Balshem et al., 2011). The working group decided to start the rating for air pollution observational studies at moderate certainty evidence rather than high, because of the risk of unmeasured confounding in observational research. The certainty of the evidence from this level was then downgraded or upgraded, based on the criteria per GRADE domain. Criteria that were introduced to complement or replace the existing criteria include the following:

• the calculation of an 80 % prediction interval, to assess heterogeneity (IntHout et al., 2016);
• the calculation of the sample size needed for a study based on a specific relative risk and confidence interval, for imprecision (Rothman and Greenland, 2018);
• the estimation of the extent to which confounding may influence a pooled effect size using the E-value, for large magnitude of effect size (Mathur and VanderWeele, 2020); and
• additional approaches to help assess publication bias, such as analyses of multicentre versus single-city studies or differences in effect estimates of earlier versus later studies.

## 7 From evidence to recommendations

The evidence on adverse health effects synthesized in this Special Issue is the basis of the formulation of the new air quality guideline levels. Each of the systematic reviews provides a summary estimate of the relative risk derived from the *meta*-analyses for each air pollutant–outcome pair, a 95 % confidence interval around that estimate, and qualification of the certainty of the evidence. Transparent procedures to move from the systematic review evidence to air quality guideline levels were developed by a dedicated working group; the methodological approach is described in detail in the guideline document (WHO, 2021).

As mentioned above, additional guidance in the form of interim targets and good practice statements about certain types of PM has also been provided. Further, a dedicated chapter on implementation of the guidelines offers information on air quality management, as well as guidance on health impact assessment and setting air quality standards and policies.

## 8 Other supporting evidence

This Special Issue also contains papers, commissioned within the framework of related WHO projects, that followed different approaches to the core systematic reviews of evidence presented above. These papers were discussed in the context of deciding whether to derive an AQG level or good practice statements for particles originating from sand and dust storms (the first and second reviews listed below) and in relation to certain implementation aspects (the third in the list):

• Mechanisms underlying the health effects of desert sand dust (Fussell and Kelly, 2021; this issue);
• Health effects of desert dust and sand storms (planned for this issue, protocol published as Tobias et al., 2019); and
• The effect of using personal-level indoor air cleaners and respirators on biomarkers of cardiorespiratory health: a systematic review (Liu et al., 2022; this issue).

Other WHO-commissioned papers discussed during the guideline development process in relation to implementation aspects (interim targets, air filtration) have been published elsewhere (Evangelopoulos et al., 2020, Zhu et al., 2021).

In summary, the new WHO global air quality guidelines are informed by the best available scientific evidence covering a vast number of research papers published until September 2018, and appraised by experts and stakeholders in the field of air quality. The process of synthesizing the evidence was complex and challenging, given the need to adapt methods largely devised for assessing clinical interventions. Overcoming these challenges resulted in operational methodological developments applicable for the air quality guidelines. Research gaps remain and, therefore, recommendations for future research are formulated in the guideline document. These include the need for the expert community to continue working to further improve the methods for evidence synthesis and evaluation in this field. At a more general level, there is a need to advance the policy-relevant scientific base and to enhance evidence supporting policy decisions worldwide, especially in LMICs. Many LMICs still lack the resources to monitor the most common air pollutants and conduct research on their adverse effects on health. All in all, the new guidelines are expected to drive countries’ efforts to reduce population exposure to air pollution and come closer to the goals of the 2030 Agenda for Sustainable Development.

## 9 Disclaimer

The authors alone are responsible for the views expressed in this publication, and they do not necessarily represent the decisions or the stated policy of WHO.

## Declaration of Competing Interest

The authors declare that they have no known competing financial interests or personal relationships that could have appeared to influence the work reported in this paper.

## Data Availability

No data was used for the research described in the article.

## References

[b0005] Awe, Y., Hagler, G., Kleiman, G., Klopp, J., Pinder, R., Terry, S., 2017. Filling the gaps: Improving measurement of ambient air quality in low and middle income countries. White paper. Discussion draft.

[b0010] Balshem H., Helfand M., Schünemann H.J., Oxman A.D., Kunz R., Brozek J., Vist G.E., Falck-Ytter Y., Meerpohl J., Norris S., Guyatt G.H. (2011). GRADE guidelines: 3. Rating the quality of evidence. J. Clin. Epidemiol..

[b0015] Bazyar J., Pourvakhshoori N., Khankeh H., Farrokhi M., Delshad V., Rajabi E. (2019). A comprehensive evaluation of the association between ambient air pollution and adverse health outcomes of major organ systems: a systematic review with a worldwide approach. Environ. Sci. Pollut. Res..

[b0020] Campbell-Lendrum D., Prüss-Ustün A. (2019). Climate change, air pollution and noncommunicable diseases. Bull. World Health Organ..

[b0025] Chen J., Hoek G. (2020). Long-term exposure to PM and all-cause and cause-specific mortality: a systematic review and meta-analysis. Environ. Int..

[b0030] Cohen, A.J., Samet, J.M., Straif, K., IARC, 2013. Air pollution and cancer. International Agency for Research on Cancer, Lyon.

[b0035] Cromar K.R., Duncan B.N., Bartonova A., Benedict K., Brauer M., Habre R., Hagler G.S.W., Haynes J.A., Khan S., Kilaru V., Liu Y., Pawson S., Peden D.B., Quint J.K., Rice M.B., Sasser E.N., Seto E., Stone S.L., Thurston G.D., Volckens J. (2019). Air pollution monitoring for health research and patient care. An Official American Thoracic Society Workshop Report. Annals of the American Thoracic Society.

[b0040] Evangelopoulos D., Perez-Velasco R., Walton H., Gumy S., Williams M., Kelly F.J., Künzli N. (2020). The role of burden of disease assessment in tracking progress towards achieving WHO global air quality guidelines. Int. J. Public Health.

[b0045] Fussell J.C., Kelly F.J. (2021). Mechanisms underlying the health effects of desert sand dust. Environ. Int..

[b0050] Huangfu P., Atkinson R. (2020). Long-term exposure to NO2 and O3 and all-cause and respiratory mortality: a systematic review and meta-analysis. Environ. Int..

[b0055] Hultcrantz M., Rind D., Akl E.A., Treweek S., Mustafa R.A., Iorio A., Alper B.S., Meerpohl J.J., Murad M.H., Ansari M.T., Katikireddi S.V., Östlund P., Tranæus S., Christensen R., Gartlehner G., Brozek J., Izcovich A., Schünemann H., Guyatt G. (2017). The GRADE Working Group clarifies the construct of certainty of evidence. J. Clin. Epidemiol..

[b0060] IntHout J., Ioannidis J.P.A., Rovers M.M., Goeman J.J. (2016). Plea for routinely presenting prediction intervals in meta-analysis. BMJ Open.

[b0065] Kelly F.J., Fussell J.C. (2015). Air pollution and public health: emerging hazards and improved understanding of risk. Environ. Geochem. Health.

[b0070] Krzyzanowski M., Cohen A. (2008). Update of WHO air quality guidelines. Air Qual. Atmos. Health.

[b0075] Lee K.K., Spath N., Miller M.R., Mills N.L., Shah A.S.V. (2020). Short-term exposure to carbon monoxide and myocardial infarction: a systematic review and meta-analysis. Environ. Int..

[b0080] Liu S., Wu R., Zhu Y., Wang T., Fang J., Xie Y., Yuan N., Xu H., Song X., Huang W. (2022). The effect of using personal-level indoor air cleaners and respirators on biomarkers of cardiorespiratory health: a systematic review. Environ Int..

[b0085] Longhurst, J., Barnes, J., Chatterton, T., et al., 2018. Analysing air pollution and its management through the lens of the UN Sustainable Development Goals: a review and assessment, in: Casares, J., Passerini, G., Barnes, J., et al. (Ed.), Air Pollution XXVI, WIT Transactions on Ecology and the Environment. WIT Press, Ashurst Lodge.

[b0090] Mathur M.B., VanderWeele T.J. (2020). Sensitivity analysis for unmeasured confounding in meta-analyses. J. Am. Stat. Assoc..

[b0095] Morgan R.L., Thayer K.A., Bero L., Bruce N., Falck-Ytter Y., Ghersi D., Guyatt G., Hooijmans C., Langendam M., Mandrioli D., Mustafa R.A., Rehfuess E.A., Rooney A.A., Shea B., Silbergeld E.K., Sutton P., Wolfe M.S., Woodruff T.J., Verbeek J.H., Holloway A.C., Santesso N., Schünemann H.J. (2016). GRADE: Assessing the quality of evidence in environmental and occupational health. Environ. Int..

[b0100] Morgan R.L., Whaley P., Thayer K.A., Schünemann H.J. (2018). Identifying the PECO: A framework for formulating good questions to explore the association of environmental and other exposures with health outcomes. Environ. Int..

[b0105] Morgan R.L., Thayer K.A., Santesso N., Holloway A.C., Blain R., Eftim S.E., Goldstone A.E., Ross P., Ansari M., Akl E.A., Filippini T., Hansell A., Meerpohl J.J., Mustafa R.A., Verbeek J., Vinceti M., Whaley P., Schünemann H.J. (2019). A risk of bias instrument for non-randomized studies of exposures: A users’ guide to its application in the context of GRADE. Environ. Int..

[b0110] Orellano P., Reynoso J., Quaranta N., Bardach A., Ciapponi A. (2020). Short-term exposure to particulate matter (PM10 and PM2.5), nitrogen dioxide (NO2), and ozone (O3) and all-cause and cause-specific mortality: Systematic review and meta-analysis. Environ. Int..

[b0115] Orellano P., Reynoso J., Quaranta N. (2021). Short-term exposure to sulphur dioxide (SO_2_) and all-cause and respiratory mortality: a systematic review and meta-analysis. Environ. Int..

[b0120] Page M.J., Shamseer L., Tricco A.C. (2018). Registration of systematic reviews in PROSPERO: 30,000 records and counting. Systematic Rev..

[b0125] Rothman K., Greenland S. (2018). Planning study size based on precision rather than power. Epidemiology.

[b0130] Shaddick G., Thomas M.L., Green A., Brauer M., van Donkelaar A., Burnett R., Chang H.H., Cohen A., Dingenen R.V., Dora C., Gumy S., Liu Y., Martin R., Waller L.A., West J., Zidek J.V., Prüss-Ustün A. (2018). Data integration model for air quality: a hierarchical approach to the global estimation of exposures to ambient air pollution. J. Roy. Stat. Soc.: Ser. C (Appl. Stat.).

[b0135] Tobias A., Karanasiou A., Amato F., Roqué M., Querol X. (2019). Health effects of desert dust and sand storms: a systematic review and meta-analysis protocol. BMJ Open.

[b0140] UNEA, 2016. United Nations Environment Assembly resolution 2/21, “Sand and dust storms,” UNEP/EA.2/Res.21. URL https://wedocs.unep.org/handle/20.500.11822/11194?show=full (accessed 01.08.22).

[b0145] UNEA, 2017a. United Nations Environment Assembly resolution 1/7, “Strengthening the role of the United Nations Environment Programme in promoting air quality,” UNEP/EA.1/Res.7. URL https://www.informea.org/en/decision/strengthening-role-united-nations-environment-programme-promoting-air-quality (accessed 01.08.22).

[b0150] UNEA, 2017b. United National Environment Assembly draft resolution 3/23, “Preventing and reducing air pollution to improve air quality globally,” UNEP/EA.3/L.23. URL https://wedocs.unep.org/handle/20.500.11822/31023 (accessed 01.08.22).

[b0155] UNEP, 2017. Monitoring air quality [WWW Document]. URL http://www.unenvironment.org/explore-topics/air/what-we-do/monitoring-air-quality (accessed 04.05.22).

[b0160] UNGA, 2015. Transforming our world: the 2030 Agenda for Sustainable Development, A/RES/70/1. URL https://www.un.org/en/development/desa/population/migration/generalassembly/docs/globalcompact/A_RES_70_1_E.pdf (accessed 01.08.22).

[b0165] UNGA, 2018. Political declaration of the 3rd High-Level Meeting of the General Assembly on the Prevention and Control of Non-Communicable Diseases: resolution/adopted by the General Assembly, A/RES/73/2. URL https://digitallibrary.un.org/record/1648984/files/A_RES_73_2-EN.pdf?ln=en (accessed 01.08.22).

[b0170] UNGA, 2019. Report of the Special Rapporteur, “Issue of human rights obligations relating to the enjoyment of a safe, clean, healthy and sustainable environment”, A/HRC/40/55. URL https://undocs.org/en/A/HRC/40/55 (accessed 01.08.22).

[b0175] Vallero, D.A., 2014. Fundamentals of air pollution. Academic Press, Waltham.

[b0180] West J.J., Cohen A., Dentener F., Brunekreef B., Zhu T., Armstrong B., Bell M.L., Brauer M., Carmichael G., Costa D.L., Dockery D.W., Kleeman M., Krzyzanowski M., Künzli N., Liousse C., Lung S.-C., Martin R.V., Pöschl U., Pope C.A., Roberts J.M., Russell A.G., Wiedinmyer C. (2016). What we breathe impacts our health: improving understanding of the link between air pollution and health. Environ. Sci. Technol..

[b0185] Whaley, P., Nieuwenhuijsen, M., Burns, J., editors, 2021. Update of the WHO global air quality guidelines: systematic reviews. Environ Int. 142 (Special Issue) URL https://www.sciencedirect.com/journal/environment-international/special-issue/10MTC4W8FXJ (accessed 01.08.22).

[b0190] WHO Global Air Quality Guidelines Working Group on Certainty of Evidence Assessment, 2020. Approach to assessing the certainty of evidence from systematic reviews informing WHO global air quality guidelines. [WWW Document]. URL https://ars.els-cdn.com/content/image/1-s2.0-S0160412020318316-mmc4.pdf (accessed 04.05.22) (this issue).

[b0195] WHO Global Air Quality Guidelines Working Group on Risk of Bias Assessment, 2020. Risk of bias assessment instrument for systematic reviews informing WHO global air quality guidelines. Copenhagen: WHO Regional Office for Europe [WWW Document]. URL https://apps.who.int/iris/handle/10665/341717 (accessed 04.05.22).

[b0200] WHO Regional Office for Europe, 1980. Glossary on air pollution. World Health Organization. Regional Office for Europe, Copenhagen. URL https://apps.who.int/iris/handle/10665/272866 (accessed 01.08.22).

[b0205] WHO Regional Office for Europe, 1987. Air quality guidelines for Europe. WHO Regional Office for Europe, Copenhagen. URL https://apps.who.int/iris/handle/10665/107364 (accessed 01.08.22).

[b0210] WHO Regional Office for Europe, 1999. Monitoring ambient air quality for health impact assessment. WHO Regional Office for Europe, Copenhagen. URL https://apps.who.int/iris/handle/10665/107332 (accessed 01.08.22). 10791104

[b0215] WHO Regional Office for Europe, 2000. Air quality guidelines for Europe. 2^nd^ edition. WHO Regional Office for Europe, Copenhagen. URL https://apps.who.int/iris/handle/10665/107335 (accessed 01.08.22).

[b0220] WHO Regional Office for Europe, 2006. Air quality guidelines global update 2005: particulate matter, ozone, nitrogen dioxide and sulfur dioxide. WHO Regional Office for Europe, Copenhagen. URL https://apps.who.int/iris/handle/10665/107823 (accessed 01.08.22).

[b0225] WHO Regional Office for Europe, 2009. WHO guidelines for indoor air quality: dampness and mould. WHO Regional Office for Europe, Copenhagen. URL https://apps.who.int/iris/handle/10665/164348 (accessed 01.08.22).

[b0230] WHO Regional Office for Europe, 2010. WHO guidelines for indoor air quality: selected pollutants. World Health Organization. Regional Office for Europe. URL https://apps.who.int/iris/handle/10665/260127 (accessed 01.08.22). 23741784

[b0235] WHO Regional Office for Europe, 2013. Review of evidence on health aspects of air pollution – REVIHAAP Project: Technical Report. WHO Regional Office for Europe, Copenhagen. URL https://apps.who.int/iris/handle/10665/341712 (accessed 01.08.22). 27195369

[b0240] WHO Regional Office for Europe, 2017. Evolution of WHO air quality guidelines: past, present and future. World Health Organization. Regional Office for Europe, Copenhagen. URL https://apps.who.int/iris/handle/10665/341912 (accessed 01.08.22).

[b0245] WHO, 2009. Global health risks: mortality and burden of disease attributable to selected major risks. World Health Organization, Geneva, Switzerland. URL https://apps.who.int/iris/handle/10665/44203 (accessed 01.08.22).

[b0250] WHO, 2014a. WHO indoor air quality guidelines: household fuel combustion. World Health Organization, Geneva. URL https://apps.who.int/iris/handle/10665/141496 (accessed 01.08.22). 25577935

[b0255] WHO, 2014b. WHO handbook for guideline development, 2nd ed. World Health Organization, Geneva. URL https://apps.who.int/iris/handle/10665/145714 (accessed 01.08.22).

[b0260] WHO, 2015. Reducing global health risks through mitigation of short-lived climate pollutants. Scoping report for policy-makers. World Health Organization, Geneva. URL https://apps.who.int/iris/handle/10665/189524 (accessed 01.08.22).

[b0265] WHO, 2016a. Ambient air pollution: a global assessment of exposure and burden of disease. World Health Organization, Geneva. URL https://apps.who.int/iris/handle/10665/250141 (accessed 01.08.22).

[b0270] WHO, 2016b. Health and the environment: draft road map for an enhanced global response to the adverse health effects of air pollution: report by the Secretariat. World Health Organization, Geneva. URL https://apps.who.int/iris/handle/10665/250653 (accessed 01.08.22).

[b0275] WHO, 2018a. Air pollution and child health: prescribing clean air: summary. World Health Organization, Geneva. URL https://apps.who.int/iris/handle/10665/275545 (accessed 01.08.22).

[b0280] WHO, 2018b. Clean Air for Health: Geneva Action Agenda. First WHO Global Conference on Air Pollution and Health – summary report [WWW Document]. URL https://www.who.int/news/item/01-11-2018-clean-air-for-health-geneva-action-agenda (accessed 04.05.22).

[b0285] WHO, 2018c. Global database of household air pollution measurements [WWW Document]. URL https://www.who.int/data/gho/data/themes/air-pollution/hap-measurement-db#:∼:text=The%20Global%20database%20of%20household%20air%20pollution%20measurements%2C,contains%20measurements%20from%20196%20studies%20from%2043%20countries (accessed 04.05.22).

[b0290] WHO, 2018d. Burden of disease from ambient air pollution for 2016. v2. April 2018. URL https://cdn.who.int/media/docs/default-source/air-pollution-documents/air-quality-and-health/aap_bod_results_may2018_final.pdf (accessed 01.08.22).

[b0295] WHO, 2018e. Burden of disease from household air pollution for 2016. v3. April 2018. URL https://cdn.who.int/media/docs/default-source/air-pollution-documents/air-quality-and-health/hap_bod_results_may2018_final.pdf (accessed 01.08.22).

[b0300] WHO, 2018f. Burden of disease from the joint effects of household and ambient air pollution for 2016. v2 May 2018. URL https://cdn.who.int/media/docs/default-source/air-quality-database/aqd-2018/ap_joint_effect_bod_results_may2018.pdf?sfvrsn=4dc44c26_3 (accessed 01.08.22).

[b0305] WHO, 2021. WHO global air quality guidelines: particulate matter (PM2.5 and PM10), ozone, nitrogen dioxide, sulfur dioxide and carbon monoxide. World Health Organization, Geneva. URL https://apps.who.int/iris/handle/10665/345329 (accessed 01.08.22). 34662007

[b0310] WHO, 2022a. Ambient air pollution: policy and progress [WWW Document]. URL https://www.who.int/teams/environment-climate-change-and-health/air-quality-and-health/ambient-air-pollution/policy-and-progress (accessed 04.05.22).

[b0315] WHO, 2022a. Ambient air pollution: policy and progress [WWW Document]. URL https://www.who.int/teams/environment-climate-change-and-health/air-quality-and-health/ambient-air-pollution/policy-and-progress (accessed 04.05.22).

[b0320] Woodruff T.J., Sutton P. (2011). An evidence-based medicine methodology to bridge the gap between clinical and environmental health sciences. Health Aff..

[b0325] World Bank, IHME, 2016. The cost of air pollution: strengthening the economic case for action (No. 108141). The World Bank, Washington, DC.

[b0330] World Health Assembly, 2015. World Health Assembly resolution 68/8, “Health and the environment: addressing the health impact of air pollution”, WHA68.8.

[b0335] Zheng X.Y., Orellano P., Lin H.L., Jiang M., Guan W.J. (2021). Short-term exposure to ozone, nitrogen dioxide, and sulphur dioxide and emergency department visits and hospital admissions due to asthma: A systematic review and meta-analysis. Environ. Int..

[b0340] Zhu Y., Song X., Wu R., Fang J., Liu L., Wang T., Liu S., Xu H., Huang W. (2021). A review on reducing indoor particulate matter concentrations from personal-level air filtration intervention under real-world exposure situations. Indoor Air.

